# Sporotrichosis in the nasal mucosa: A single-center retrospective study of 37 cases from 1998 to 2020

**DOI:** 10.1371/journal.pntd.0011212

**Published:** 2023-03-27

**Authors:** Carlos Felipe Gomes Izoton, Antonio Xavier de Brito Sousa, Cláudia Maria Valete, Armando de Oliveira Schubach, Anna Carolina Procópio-Azevedo, Rosely Maria Zancopé-Oliveira, Priscila Marques de Macedo, Maria Clara Gutierrez-Galhardo, Julio Castro-Alves, Rodrigo Almeida-Paes, Ana Cristina da Costa Martins, Dayvison Francis Saraiva Freitas

**Affiliations:** 1 Instituto Nacional de Infectologia Evandro Chagas, Fundação Oswaldo Cruz, Rio de Janeiro, Brazil; 2 Instituto de Educação Médica, Rio de Janeiro, Brazil; 3 Faculdade de Medicina da Universidade Federal do Rio de Janeiro, Rio de Janeiro, Brazil; Albert Einstein College of Medicine, UNITED STATES

## Abstract

**Background:**

Sporotrichosis is a subcutaneous or implantation mycosis caused by some species of the genus *Sporothrix*. Rio de Janeiro state, Brazil, experiences hyperendemic levels of zoonotic sporotrichosis, with increasing cases of disseminated disease, especially in people living with HIV (PLHIV). Involvement of the nasal mucosa is rare and occurs isolated or in disseminated cases, with a delayed resolution.

**Methodology/Principal findings:**

This study aimed to describe the epidemiological, clinical, and therapeutic profiles of 37 cases of sporotrichosis with involvement of the nasal mucosa treated at the ear, nose, and throat (ENT) outpatient clinic of the Instituto Nacional de Infectologia Evandro Chagas, Fundação Oswaldo Cruz, from 1998 to 2020. Data were reviewed from the medical records and stored in a database. The Mann–Whitney test was used to compare the means of quantitative variables, and Pearson chi-square and Fisher’s exact tests were used to verify the association between qualitative variables (p<0.05). Most patients were males, students or retirees, with a median age of 38 years, residents in the municipality of Rio de Janeiro, and infected through zoonotic transmission. Disseminated sporotrichosis forms in patients with comorbidities (mostly PLHIV) were more common than the isolated involvement of the mucosa. The main characteristics of lesions in the nasal mucosa were the presence/elimination of crusts, involvement of various structures, mixed appearance, and severe intensity. Due to therapeutic difficulty, itraconazole was combined with amphotericin B and/or terbinafine in most cases. Of the 37 patients, 24 (64.9%) healed, with a median of 61 weeks of treatment, 9 lost follow-up, 2 were still treating and 2 died.

**Conclusions:**

Immunosuppression was determinant to the outcome, with worse prognosis and lower probability of cure. Notably in this group, the systematization of the ENT examination for early identification of lesions is recommended to optimize the treatment and outcome of the disease.

## Author summary

Sporotrichosis is a subcutaneous or implantation mycosis with hyperendemic levels of zoonotic transmission in Rio de Janeiro state, Brazil. The involvement of the nasal mucosa is uncommon and may occur in isolated or disseminated forms of sporotrichosis, but the number of cases has been increasing. We analyzed 37 cases of sporotrichosis with involvement of the nasal mucosa treated at a reference center for infectious diseases. There was a predominance of males, students and retirees, residents in the municipality of Rio de Janeiro, and infected by cats. The median age was 38 years. Disseminated forms of sporotrichosis predominated over isolated involvement of the nasal mucosa. The number of patients with HIV infection also caught our attention. The main characteristics of the nasal mucosa lesions were the presence/elimination of crusts, with an involvement of various structures. Most cases were treated with a combination of antifungal agents and were cured, and two deaths were observed. Immunosuppression directly influenced the prognosis of cure of nasal mucosa sporotrichosis.

## Introduction

Sporotrichosis is a worldwide subcutaneous or implantation mycosis of humans and other mammals. It is endemic in many regions of Latin America and Asia, caused by dimorphic human pathogenic species of the genus *Sporothrix*. Classically, infection occurs by environmental transmission after traumatic inoculation with soil, plants and organic matter contaminated with fungi [1]. However, since 1998, there has been an increase in human sporotrichosis cases in Brazil, particularly in the metropolitan region of the state of Rio de Janeiro, which is experiencing hyperendemic levels of zoonotic transmission of the species *Sporothrix brasiliensis*, the most virulent of the genus [1,2]. An infected cat spreads the disease through bites, scratches or its secretions and exudates [2,3]. The social problems of the country, the lack of an adequate health policy for the control of the hyperendemic [4] and the poor understanding of this disease by those affected [5] contribute to the aggravation of this scenario.

The most common clinical forms of the disease are lymphocutaneous and fixed cutaneous [6]. The disseminated form is uncommon but more severe, can affect multiple organs and systems and is primarily related to immunosuppression (e.g., HIV) [7–12]. There are also cases of disseminated sporotrichosis as a manifestation of immune reconstitution inflammatory syndrome (IRIS) after the initiation of antiretroviral therapy in people living with HIV (PLHIV) [13]. The increase in the occurrence of cases of disseminated sporotrichosis in PLHIV presumes an association between these two diseases that concerns health professionals due to the severity that both infections can assume [10,14,15].

The mucosal form is considered by some professionals a variant of the cutaneous form [16]. In its clinical classification, it can be named as mucocutaneous [17], mucosal [6,16–18] or extracutaneous/disseminated forms [8,10,11] and may appear alone or as a more extensive involvement affecting other body sites. Mucosal involvement may occur by contact of hands contaminated with the fungus, by hematogenous route, by contiguity from the skin, by inhalation of conidia (of environmental origin) [16] or yeasts (aerosols from sneezing or movements of sick cats) [3]. The ocular mucosa is the most frequently affected, followed by the nasal mucosa, which is uncommon. The most involved site of the nasal mucosa is the septum. The lesion of the mucosa has an aspect suggestive of microabscesses (Martins, ACC—personal observation), with infiltration and often associated with nasal crusts and elimination of bloody secretions [16]. Involvement of the upper aerodigestive tract mucosa is uncommon in sporotrichosis, with few reports in the literature [2,8,9,14,19–21]. In general, the resolution of cases takes longer than that of skin lesions [8,9,14,19].

The treatment of sporotrichosis is preferably performed with antifungal drugs, especially itraconazole (1st choice), terbinafine and amphotericin B [22], considering the clinical form and host immunity [23]. The duration of the treatment is until the complete remission of the signs and symptoms, being in general of three to four months, but in some cases it can be superior to one year [23]. Other treatments include cryosurgery with liquid nitrogen spray [24], local heat [25], electrosurgery, and surgical excision of the lesions [26], depending on the site and/or clinics.

The present study aimed to describe the epidemiological, clinical, and therapeutic profiles of cases of sporotrichosis with involvement of the nasal mucosa confirmed by culture and treated at the ear, nose, and throat (ENT) outpatient clinic of the Instituto Nacional de Infectologia Evandro Chagas of the Fundação Oswaldo Cruz (INI/FIOCRUZ) from 1998 to 2020.

## Methods

### Ethics statement

The cases analyzed were included based on two studies approved by the Institutional Review Board (IRB) of Instituto Nacional de Infectologia Evandro Chagas, Fundação Oswaldo Cruz (#03967512.3.0000.5262 and #40049720.0.0000.5262). The accessible patients signed an informed consent form, while the others were exempted by the IRB. The researchers signed a Term of Commitment and Responsibility, assuring confidentiality of the data.

### Study location

INI/FIOCRUZ is a public institution located in Rio de Janeiro, Brazil, and national reference in the treatment of infectious diseases, with recognized clinical and laboratory infrastructure. The ENT outpatient clinic of INI/FIOCRUZ is part of this structure and is responsible for the care of patients with ENT manifestations of infectious diseases, including sporotrichosis.

### Study design

For this study, we retrospectively assessed the case series of sporotrichosis in the nasal mucosa of the ENT clinic of INI/FIOCRUZ seen from 1998 to 2020. The records of the Laboratory of Mycology and of the ENT outpatient clinic, both from INI/FIOCRUZ, were used.

The inclusion criteria were patients who had nasal mucosa impairment and the isolation of *Sporothrix* spp. in culture from nasal swab/scraping or biopsy.

### Diagnosis of sporotrichosis in the nasal mucosa

At INI/FIOCRUZ, all patients with disseminated forms of sporotrichosis or those with nasal complaints are referred for ENT evaluation. The cases included in this study underwent endoscopic examination of the nasal cavities and collection of biological material from the mucosal lesion by swab, scraping or biopsy. The samples were sent for mycological examination and, in some cases, histopathological examination.

For the mycological examination, the samples were subjected to direct microscopy with 10% potassium hydroxide and cultures were made on BBL Sabouraud dextrose agar 2% (Becton Dickinson Co., Sparks, MA, USA) and Mycosel Agar (Becton Dickinson Co., Sparks, MA, USA) with incubation at 25°C for up to four weeks. Dimorphism of suspected *Sporothrix* colonies was assessed on brain heart infusion agar (Becton Dickinson Co., Sparks, MA, USA) at 35°C for seven days. Molecular identification of *Sporothrix* spp. strains isolated from the included patients was performed using a species-specific polymerase chain reaction protocol [27] on those cultures that were preserved in the Laboratory of Mycology and were viable at the time of molecular evaluation.

For the histopathological examination, the histological sections of the paraffinized samples were stained by hematoxylin and eosin (H&E), periodic acid-Schiff (PAS) and silver (Grocott-Gomori) impregnation.

Radiographs and/or computed tomography (CT) of the skull, neck and/or paranasal sinuses (PNS) were requested to assess the extent of the lesion.

### Definition of the clinical form of sporotrichosis located in the nasal mucosa

In addition to the clinical forms of sporotrichosis traditionally described in the literature (fixed cutaneous, disseminated cutaneous, disseminated, and lymphocutaneous), the classification “localized nasal mucosa” was adopted. This classification included cases that, in addition to the involvement of the nasal mucosa, exhibited contiguous lesions (e.g., nodular lymphangitis on the face, with or without regional lymph node enlargement) or reactive lesions (e.g., erythema nodosum).

### Classification of nasal mucosa lesions

The lesions were classified by nasal endoscopic examination according to the affected structure: septum, turbinates or diffuse (involvement of more than one structure); appearance: infiltrative, granular with and without microabscesses, crusted and mixed (when more than one aspect was observed); and intensity: mild (infiltrative and/or granular lesion without microabscess), moderate (presence of bleeding and/or granular lesion with microabscess) or severe (diffuse infiltration, multiple structures affected, crusted/mixed lesion or septal perforation).

### Treatment of sporotrichosis in the nasal mucosa

The treatment was an initial dose of 100–200 mg/day of oral itraconazole. In the absence of clinical improvement in the interval between monthly visits, the dose was increased to 400 mg/day. In cases of intolerance to itraconazole or in the absence of any improvement (drug failure), a new sample was collected for mycological examination and treatment was changed to oral terbinafine, 250–500 mg/day.

The term “combination of antifungals” was used for cases treated with itraconazole 100–400 mg/day that were supplemented by amphotericin B and/or terbinafine.

In addition to the systemic treatment, the patients were instructed to thoroughly wash (eight to ten times per day) their nasal cavities with 0.9% NaCl solution, injected under pressure through a 20 ml syringe, to keep the nasal mucosa humidified and to reduce the formation of crusts.

### Analysis of collected data

All data were collected from the review of medical records, and digitally tabulated in Microsoft Excel 2013 software. The variables studied were sex, age, place of residence, profession/occupation, likely mode of transmission, clinical form, ENT signs and symptoms, characteristics of nasal lesions (affected structure, number of affected nasal structures, appearance, and intensity), comorbidities, material collected for diagnosis, evolution in the observed period (follow-up), drugs used in treatment and treatment time. The comorbidities heart disease, alcohol consumption, human immunodeficiency virus (HIV) infection/acquired immunodeficiency syndrome (AIDS), chronic renal failure, sarcoidosis and renal transplantation were considered immunosuppression.

For the exploratory analysis, frequencies and percentages were computed for qualitative variables, and summary measures, such as median and interquartile range (IQR), were computed for quantitative variables. The comparison of the medians of the quantitative variables according to categorical variables was performed using the nonparametric Mann–Whitney test. To assess the association between qualitative variables, the Pearson chi-square test or Fisher’s exact test was used when necessary. Cox models were used to estimate HR (hazard ratio) in univariate models and aHR (adjusted hazard ratio) for adjusted models. The following variables were used: a) outcome (cure, under treatment, death, loss of follow-up): nasal mucosa (localized), other sites and sporotrichosis (all affected sites); b) exposure: clinical forms (localized versus disseminated—nasal mucosa lesion associated with other sites), immunosuppression (yes/no), and HIV infection (yes/no); and c) adjustment (control): age, sex, septal perforation, and number of affected nasal structures (**S1 Table**).

The terms “not available”, “no epidemiological history”, and “unknown” are considered missing data.

Kaplan–Meier curves were estimated for the outcomes and exposure variables described above. For those patients considered not cured (under treatment, death and loss of follow-up), the curve was censored at such moments (dates of analysis and last appointment). Values of p <0.05 were considered significant. All analyses were performed using the software R version 4.0.

## Results

### Demographic aspects

In the period analyzed (1998–2020), 5,393 patients were diagnosed with sporotrichosis at INI/FIOCRUZ. The first diagnosis of sporotrichosis of the nasal mucosa at INI/FIOCRUZ occurred in 2001. During this period, there were some years without cases of involvement of the nasal mucosa (**S1 Fig**). The 37 cases described and analyzed here represent 0.7% of the institutional sampling, with a mean of 1.6 cases/year.

Among the 37 cases evaluated, there was a predominance of males (54.1%) and a median age of 38 years (IQR: 33–47 years). The metropolitan region of Rio de Janeiro included almost all cases, especially the municipalities of Rio de Janeiro (capital) (59.5%), Duque de Caxias (18.9%) and Nova Iguaçu (5.4%). In the capital, there were cases mainly in the north and west zones, with nine (40.9%) cases each. Students and retirees accounted for five (13.5%) cases each, followed by the unemployed (8.1%). Other occupations with up to two (5.4%) cases were also observed (**S2 Table**).

### Probable transmission of sporotrichosis

In 28 (75.7%) cases, the probable inoculation occurred by contact with infected cats. Behaviors like sleeping together, sniffing, kissing, sharing nasal spray etc. were reported in 6 (16.2%) cases. In other 3 (8.1%) cases, it was due to manipulation of soil, plants and/or organic matter. In 6 (16.2%) cases, exposure form could not be determined by the patient’s report.

### Clinical form of sporotrichosis, comorbidities and relationship with other signs and symptoms

Data on the clinical form of sporotrichosis, comorbidities, the relationship with other signs and symptoms and the characteristics of nasal lesions regarding the affected structure, quantity, appearance, and intensity are shown in **Table 1**.

**Fig 1:**
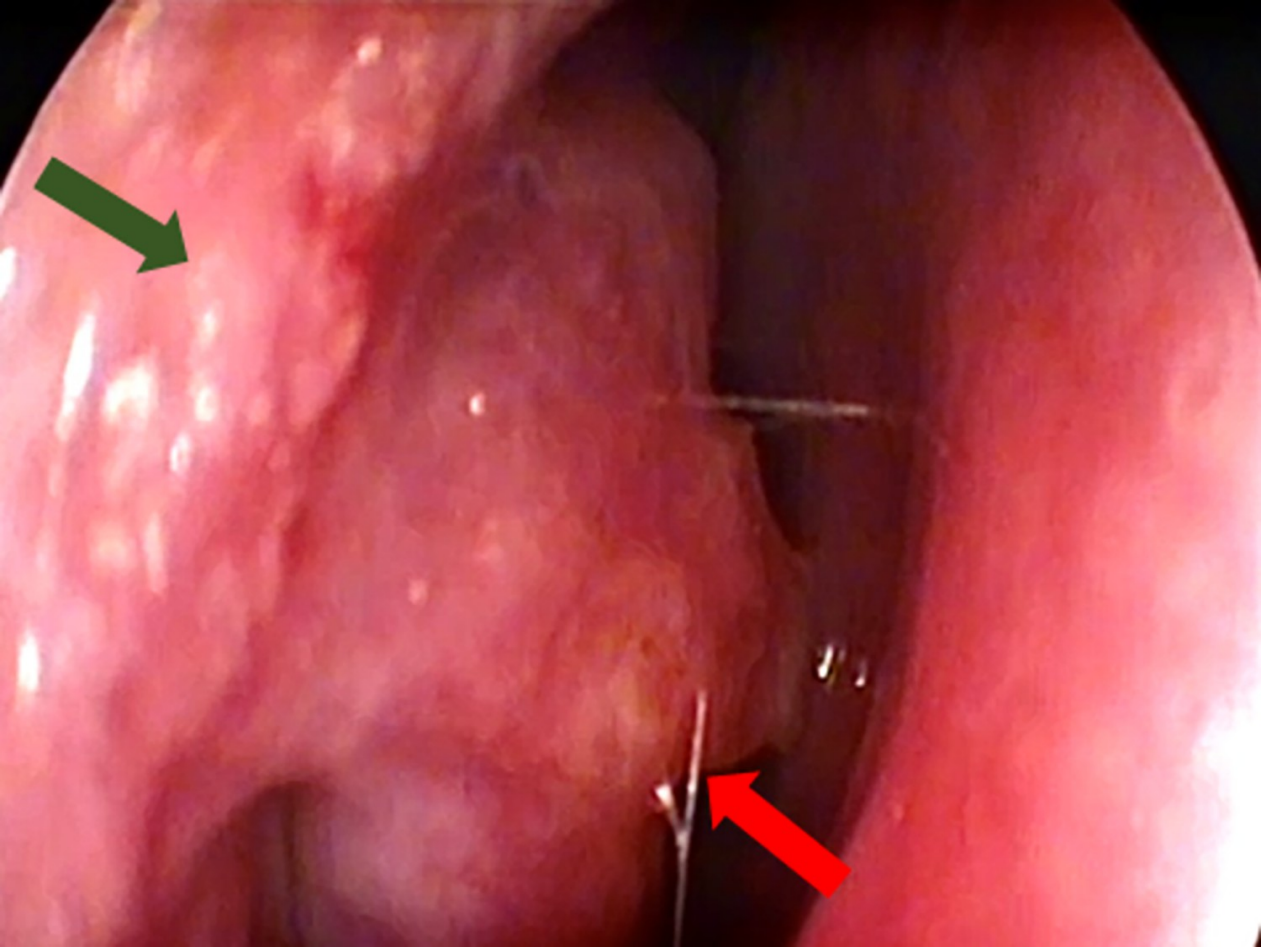
Manifestation of sporotrichosis in the nasal mucosa (isolated) of a patient (case 25) treated at the ENT outpatient clinic at INI/FIOCRUZ. Granular infiltration in the right nasal vestibule (green) and in the right inferior nasal turbinate (red). Source: Cláudia Maria Valete (ENT outpatient clinic, INI/FIOCRUZ).

**Fig 2:**
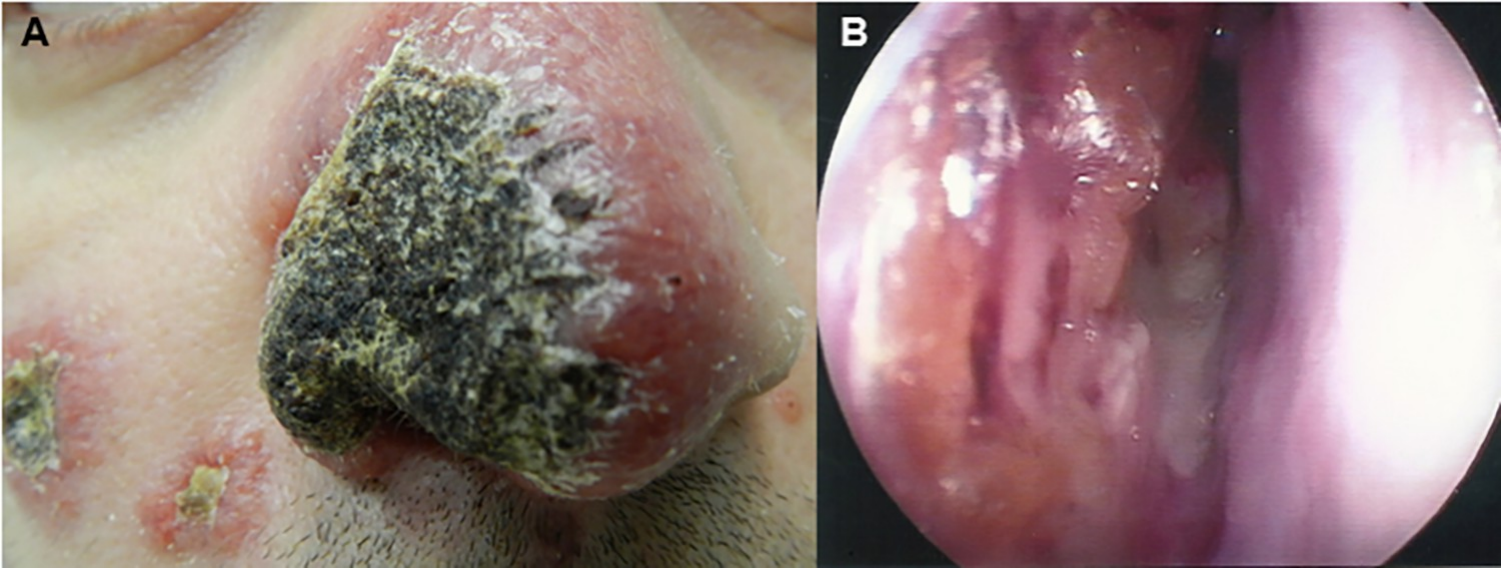
Manifestation of disseminated sporotrichosis of a patient (case 20) treated at the ENT outpatient clinic at INI/FIOCRUZ. A) Erythematous crusted lesions on the skin of the face. B) Granular infiltration and crusts in the nasal mucosa. Source: Ana Cristina da Costa Martins (ENT outpatient clinic, INI/FIOCRUZ).

In 14 (37.8%) patients, there were only manifestations in the nasal mucosa (**Fig 1**). In the remaining 23 (62.2%) patients, the involvement of the nasal mucosa was accompanied by disseminated lesions (54.1%) (**Fig 2**) and disseminated cutaneous lesions (8.1%).

**Table 1:** Clinical aspects of the 37 cases of sporotrichosis in the nasal mucosa treated at the ENT outpatient clinic of the INI/FIOCRUZ (1998–2020).

Case	Clinical form	Comorbidities	ENT signs and symptoms	Affected structure	Aspect	Intensity
1	Disseminated	HIV/AIDS, Tuberculosis	Nasal congestion / obstruction, Epistaxis, Presence / elimination of crusts	Diffuse	Mixed	Severe
2	Disseminated	Atopic dermatitis	Nasal congestion / obstruction, Epistaxis, Presence / elimination of crusts	Diffuse	Crusted	Severe
3	Disseminated cutaneous	Diabetes, Chronic renal failure, SAH, Deep vein thrombosis	Nasal congestion / obstruction	Diffuse	Infiltrative	Severe
4	Disseminated	HIV/AIDS	Rhinorrhea	Diffuse	Infiltrative	Severe
5	Disseminated cutaneous	Sarcoidosis	Bilateral involvement (RNF & LNF), Septal destruction/perforation, Mucosal infiltration, Presence/elimination of crusts, Ulceration	Septum	Mixed	Severe
6	Nasal mucosa (localized)	Nothing	Mucosal infiltration, Presence/elimination of crusts, Ulceration	Diffuse	Mixed	Severe
7	Nasal mucosa (localized)	Nothing	Presence/elimination of crusts, Rhinorrhea	Turbinates	Crusted	Severe
8	Disseminated	HIV/AIDS, Cytomegalovirus	Bilateral involvement (RNF & LNF), Nasal congestion/obstruction, Septal destruction/perforation, Pain, Mucosal infiltration, Presence/elimination of crusts, Ulceration	Diffuse	Mixed	Severe
9	Nasal mucosa (localized)	Nothing	Nasal congestion/obstruction, Pain	Turbinates	Infiltrative	Mild
10	Disseminated	HIV/AIDS	Bilateral involvement (RNF & LNF), Septal destruction/perforation, Presence/elimination of crusts, Ulceration	Diffuse	Crusted	Severe
11	Disseminated	Renal transplantation	Nasal congestion/obstruction, Presence/elimination of crusts	Turbinates	Crusted	Severe
12	Disseminated	Diabetes, SAH, Chronic renal failure	Bilateral involvement (RNF & LNF), Granulation, Mucosal infiltration	Diffuse	Mixed	Severe
13	Disseminated	Heart disease, SAH, HIV/AIDS	Hyperemia, Synechia	Diffuse	Infiltrative	Severe
14	Disseminated	HIV/AIDS	Bilateral involvement (RNF & LNF), Nasal congestion/obstruction, Mucosal infiltration, Presence/elimination of crusts, Rhinorrhea	Diffuse	Mixed	Severe
15	Nasal mucosa (localized)	Nothing	Nasal congestion/obstruction, Presence/elimination of crusts	Septum	Mixed	Severe
16	Disseminated	SAH	Bilateral involvement (RNF & LNF), Nasal congestion/obstruction, Presence/elimination of crusts	Diffuse	Mixed	Severe
17	Nasal mucosa (localized)	Nothing	Presence/elimination of crusts	Septum	Mixed	Severe
18	Disseminated	Nothing	Nasal congestion/obstruction, Epistaxis	Diffuse	Mixed	Severe
19	Nasal mucosa (localized)	Nothing	Pain, Presence/elimination of crusts	Turbinates	Mixed	Severe
20	Disseminated	HIV/AIDS, IRIS	Bilateral involvement, Nasal congestion/obstruction, Fever, Rhinorrhea	Diffuse	Mixed	Severe
21	Disseminated	Chemical dependence, Alcohol abuse, HIV/AIDS	Nasal congestion/obstruction, Dysphonia, Pain, Hemoptysis, Odynophonia, Presence/elimination of crusts, Cough	Diffuse	Mixed	Severe
22	Disseminated	Nothing	Pain, Epistaxis	Turbinates	Mixed	Moderate
23	Nasal mucosa (localized)	Nothing	Pain, Epistaxis, Rhinorrhea	Septum	Infiltrative	Moderate
24	Disseminated	Alcohol abuse	Granulation with microabscesses, Mucosal infiltration, Lymph node enlargement	Septum	Mixed	Moderate
25	Nasal mucosa (localized)	Nothing	Nasal congestion/obstruction, Lymph node enlargement, Presence/elimination of crusts	Turbinates	Mixed	Severe
26	Nasal mucosa (localized)	Nothing	Rhinorrhea	Septum	Infiltrative	Mild
27	Disseminated	HIV/AIDS	Bilateral involvement (RNF & LNF), Nasal congestion/obstruction, Epistaxis, Odynophagia, Presence/elimination of crusts	Diffuse	Mixed	Severe
28	Disseminated	HIV/AIDS, Cytomegalovirus	Bilateral involvement (RNF & LNF), Nasal congestion/obstruction, Septal destruction/perforation, Presence/elimination of crusts	Septum	Crusted	Severe
29	Nasal mucosa (localized)	Nothing	Mucosal infiltration, Presence/elimination of crusts, Ulceration	Septum	Mixed	Severe
30	Disseminated	Alcohol abuse	Granulation, Hyperemia, Rhinorrhea	Diffuse	Mixed	Severe
31	Disseminated cutaneous	Nothing	Presence/elimination of crusts	Diffuse	Mixed	Severe
32	Disseminated	SAH, HIV/AIDS	Nasal congestion/obstruction, Pain, Epistaxis, Mucosal infiltration, Pyrosis, Presence/elimination of crusts	Diffuse	Mixed	Severe
33	Disseminated	HIV/AIDS, Neurotoxoplasmosis	Septal destruction/perforation, Mucosal infiltration, Rhinorrhea	Septum	Mixed	Severe
34	Nasal mucosa (localized)	Heart disease	Granulation, Hyperemia, Mucosal infiltration	Septum	Mixed	Mild
35	Nasal mucosa (localized)	Respiratory disorders	Presence/elimination of crusts	Septum	Mixed	Severe
36	Nasal mucosa (localized)	Nothing	Pain, Granulation with microabscesses	Diffuse	Granular	Severe
37	Nasal mucosa (localized)	Nothing	Bilateral involvement (RNF & LNF), Hyperemia, Dryness	Diffuse	Mixed	Severe

RNF: right nasal fossae; LNF: left nasal fossae; SAH: systemic arterial hypertension; HIV: human immunodeficiency virus; ENT: ear, nasal, and throat; IRIS: immune reconstitution inflammatory syndrome. Source: Electronic patient data system and Laboratory of Mycology database, both from INI/FIOCRUZ.

There were comorbidities in 22 (59.5%) cases: HIV/AIDS (n = 12/22, 54.6%); hypertension (n = 5/22, 22.7%); alcohol abuse (n = 3/22, 13.6%); diabetes, heart disease, chronic renal failure, and cytomegalovirus infection (n = 2/22, 9.1%, each). Other diseases mentioned were respiratory disorders, chemical dependence, atopic dermatitis, sarcoidosis, IRIS, neurotoxoplasmosis, renal transplantation, deep vein thrombosis and tuberculosis in one (4.6%) case each. Ten (27%) of these patients had more than one comorbidity, and nineteen (51.4%) were considered immunosuppressed. In 15 (40.5%) cases, there were no comorbidities.

The most reported nasal signs and symptoms were the presence/elimination of crusts (56.8%); nasal congestion/obstruction (43.2%); mucosal infiltration (27%); bilateral involvement of the nasal cavities (27%); pain and rhinorrhea (21.6%); epistaxis (19%); granulation of the mucosal surface (16.2%); ulceration and septal destruction/perforation (13.5%); hyperemia (10.8%); and granulation with microabscesses and lymph node enlargement (5.4%). Dysphonia, fever, hemoptysis, odynophagia, odynophonia, mucosal dryness, synechia and cough accounted for one (2.7%) case each. Most of these patients (83.8%) had more than one sign or symptom.

### Affected nasal structure and number of lesions

The nasal septum was the only site in 11 (29.7%) cases, followed by the nasal turbinates in six (16.2%). In 20 (54.1%) cases, the lesion was diffuse, i.e., it affected several structures simultaneously. A single nasal lesion was observed in 18 (48.7%) cases, while in 19 (51.4%) cases multiple lesions were observed.

### Appearance and intensity of nasal lesions

**Fig 3:**
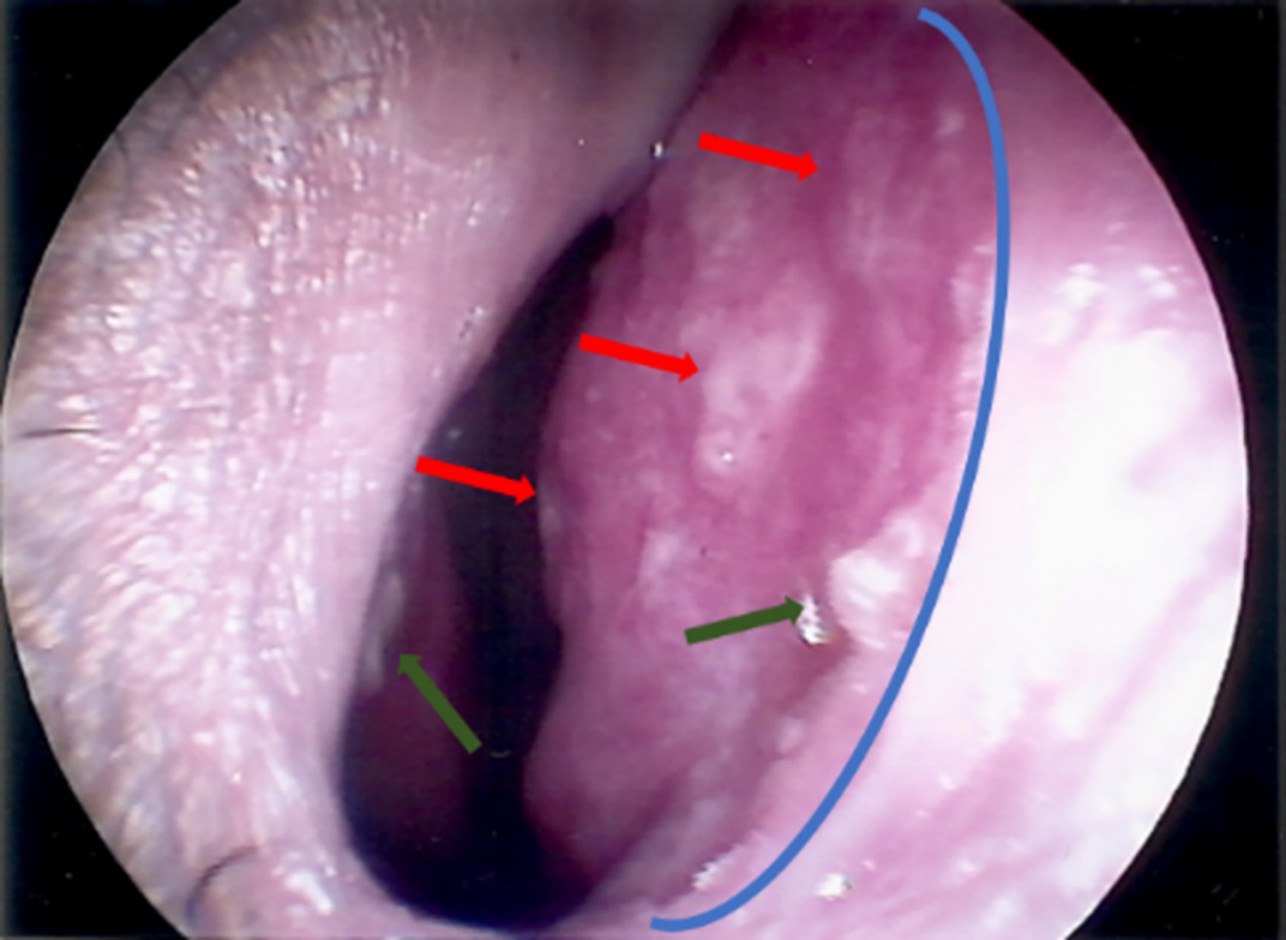
Mixed appearance of sporotrichosis in the nasal mucosa of a patient (case 24) treated at the ENT outpatient clinic at INI/FIOCRUZ. Nasal septum (right). Infiltration (blue). Granulation (red). Microabscesses (green). Source: Ana Cristina da Costa Martins (ENT outpatient clinic, INI/FIOCRUZ).

On rhinoscopy, infiltrative lesions were seen in six (16.2%) cases, followed by crusted lesions in five (13.5%) cases. The granular presentation (with or without microabscesses) was seen in one (2.7%) case. However, the mixed aspect (**Fig 3**) was predominant in 25 (67.6%) cases. Regarding the intensity, the lesions were classified as severe in 31 (83.8%) cases and as moderate or mild in three (8.1%) cases.

### Mycological and histopathological diagnosis, and molecular identification

The mycological diagnosis was obtained by culture of swab/nasal scraping in 29 (78.4%) cases and biopsy in other 8 (21.6%) cases. Chronic granulomatous inflammatory process and the presence of yeast-like fungal forms compatible with *Sporothrix* sp. by the Grocott-Gomori and PAS techniques were observed in five cases each.

Of the 37 cases, it was possible to recover 20 (54.1%) fungal isolates, all identified as *S*. *brasiliensis*. The isolates were also subjected to polymerase chain reaction with *S*. *schenckii* and *S*. *globosa* primers, with negative results (**S2 Fig**).

### Complementary diagnostic imaging

Imaging examinations of the skull, neck and/or PNS were requested in 16 (43.2%) cases, and the majority (n = 12/16; 75%) had disseminated forms of sporotrichosis. In eight (50%) cases, images with alterations in the structures of the upper aerodigestive tract were seen, highlighting the CT of PNS (n = 6/8, 75%) as the most requested examination. In these, the erosion/destruction of the bone/cartilaginous nasal septum (n = 3/6, 50%), the presence of material with soft tissue density in the nasal cavities (n = 3/6, 50%) and thickening of the maxillary sinuses (n = 2/6; 33.3%) were the most described aspects. CT also revealed thickening and irregularity of the mucosa in the nasal cavities and partial filling of the maxillary sinuses.

### Treatment: medication used, time and outcome

The combination of itraconazole 100–400 mg/day with other drugs (amphotericin B and/or terbinafine) was established in 23 (62.2%) cases, followed by the isolated use of itraconazole 100 mg/day in 11 (29.7%) cases and itraconazole 200 mg/day in 3 (8.1%). For the 25 (67.6%) patients with data on nasal mucosa healing, a median of 38 weeks (IQR: 27.1–92.7 weeks) of treatment was recorded, while for the 24 (64.9%) cured in the general context of the disease, the median recorded was 61 weeks (IQR: 25.9–102.3). At the end of the observation period, 2 (5.4%) patients remained under treatment, 9 (24.3%) were lost to follow-up, and 2 (5.4%) died due to AIDS-related complications. These data, together with information about the clinical form and the affected site, are shown in **Table 2**.

**Table 2:** Treatment and outcome of 37 cases of sporotrichosis in the nasal mucosa, according to clinical form, treated at the ENT outpatient clinic of the INI/FIOCRUZ (1998–2020).

Case	Clinical form	Other sites	Medication	Time of treatment (in weeks)^1^	Outcome
1	Disseminated^2^	Skin (multiple), larynx, bone (femur)	Antifungal combination^3^	65	Under treatment
2	Disseminated	Skin (multiple), lip, bone (tibia & fibula)	Itraconazole 200 mg	133	Cure
3	Disseminated	Skin (multiple)	Antifungal combination	114	Cure
4	Disseminated	Skin (multiple)	Antifungal combination	238	Loss of follow-up
5	Disseminated	Skin (multiple)	Itraconazole 200 mg	203	Under treatment
6	Localized	NA	Antifungal combination	40	Cure
7	Localized	NA	Itraconazole 100 mg	16	Cure
8	Disseminated	Skin (nose), CNS, bone (hand, radio, ulna)	Antifungal combination	114	Death
9	Localized	NA	Itraconazole 100 mg	34	Cure
10	Disseminated	Skin, lymph node, bone (multiple)	Antifungal combination	298	Loss of follow-up
11	Disseminated	Skin (multiple), oral mucosa	Antifungal combination	150	Cure
12	Disseminated	Skin (multiple), bone (L hand)	Antifungal combination	100	Loss of follow-up
13	Disseminated	Skin (multiple)	Antifungal combination	65	Cure
14	Disseminated	Skin (multiple), oral mucosa, eye, bone (multiple)	Antifungal combination	173	Death
15	Localized	NA	Itraconazole 200 mg	21	Cure
16	Disseminated	Skin (multiple), oral mucosa, bone (hands)	Antifungal combination	65	Cure
17	Localized	NA	Antifungal combination	13	Cure
18	Disseminated	Skin (multiple)	Antifungal combination	61	Cure
19	Localized	NA	Antifungal combination	17	Cure
20	Disseminated	Skin (multiple), bone (multiple)	Antifungal combination	458	Loss of follow-up
21	Disseminated	Skin (multiple), oropharynx, eye, bone (multiple), blood	Antifungal combination	374	Loss of follow-up
22	Disseminated	Skin (hand)	Itraconazole 100 mg	18	Cure
23	Localized	NA	Antifungal combination	30	Cure
24	Disseminated	Skin (toe)	Antifungal combination	76	Loss of follow-up
25	Localized	NA	Itraconazole 100 mg	14	Cure
26	Localized	NA	Itraconazole 100 mg	1	Loss of follow-up
27	Disseminated	Skin, oropharyngeal, and genital mucosa, synovia, bone (L foot, R knee)	Antifungal combination	574	Cure
28	Disseminated	Skin (multiple)	Antifungal combination	187	Loss of follow-up
29	Localized	NA	Itraconazole 100 mg	67	Cure
30	Disseminated	Skin (L ear)	Itraconazole 100 mg	25	Cure
31	Disseminated	Skin (R thumb, BLE), eye	Itraconazole 100 mg	20	Cure
32	Disseminated	Skin, oral mucosa, bone (multiple)	Antifungal combination	265	Cure
33	Disseminated	Oral mucosa	Antifungal combination	95	Cure
34	Localized	NA	Antifungal combination	115	Cure
35	Localized	NA	Itraconazole 100 mg	11	Cure
36	Localized	NA	Itraconazole 100 mg	26	Cure
37	Localized	NA	Itraconazole 100 mg	9	Loss of follow-up

NA: not applicable. CNS: central nervous system. L: left. R: right. BLE: both lower extremities. ^1^Period from the start to the end of medication. ^2^The disseminated and disseminated cutaneous sporotrichosis forms were considered as disseminated. ^3^Antifungal combination: itraconazole 100–400 mg/day combined with amphotericin B and/or terbinafine. Source: Electronic patient data system and Laboratory of Mycology database, both from INI/FIOCRUZ.

The use of systemic antibiotics (the most cited were amoxicillin/potassium clavulanate, sulfamethoxazole/trimethoprim and cephalexin) and/or topical nasal solutions (mainly hypertonic solutions, corticosteroids, and vasoconstrictors), previously or during the follow-up of treatment, was reported in 19 (51.4%) and 11 (29.7%) cases, respectively.

In seventeen cases, there was an interval between the initial ENT and non-ENT appointment, with a median interval of five weeks (IQR: 2.7–23.7). For the 5 (13.5%) patients with septal destruction/perforation, the median of this difference was 32 weeks (IQR: 2.7–34) versus 5 weeks (IQR: 3.1–7.5) for those who did not have this condition.

### Comparison between patients with localized nasal mucosa and patients with the form associated with other sites (disseminated)

Patients with localized nasal mucosa (37.8%) and patients with the form associated with other sites (disseminated) (62.2%) were compared (**Table 3**). In the first group, there was a predominance of younger people (median of 27.5 years; IQR: 16.2–46.2 years) and females (n = 10/14; 71.4%); only two (14.3%) had comorbidities (i.e., immunosuppression, heart disease), no cases in PLHIV, and more restricted nasal lesions (n = 11/14; 78.6%). In the second, there were older patients (median of 41 years; IQR: 37–47 years), males (n = 16/23; 69.6%), with comorbidities (n = 20/23; 87%), highlighting the high frequency of immunosuppressed patients (n = 18/23, 78.3%), mainly PLHIV (n = 12/23; 52.2%), and diffuse nasal lesions (n = 17/23, 73.9%) with multiple affected structures (n = 16/23; 69.6%).

**Table 3:** Association of selected variables with the clinical form of 37 cases of sporotrichosis in the nasal mucosa treated at the ENT outpatient clinic at the INI/FIOCRUZ (1998–2020).

	Clinical form		
Variable	Disseminated n (%)	Nasal mucosa (localized) n (%)	p-value
**Sex**			0.037
Female	7 (30.4)	10 (71.4)	
Male	16 (69.6)	4 (28.6)	
**Probable transmission**			0.527
Infected cat	16 (69.6)	12 (85.7)	
Others (non-cat)	3 (13)	0 (0)	
No epidemiological history	4 (17.4)	2 (14.3)	
**Comorbidities**			< 0.001
No	3 (13)	12 (85.7)	
Yes	20 (87)	2 (14.3)	
**Immunosuppression**			< 0.001
No	5 (21.7)	13 (92.9)	
Yes	18 (78.3)	1 (7.1)	
**HIV**			< 0.001
No	11 (47.8)	14 (100)	
Yes	12 (52.2)	0 (0)	
**Presence/elimination of crusts**			1
No	10 (43.5)	6 (42.9)	
Yes	13 (56.5)	8 (57.1)	
**Septal perforation**			0.135
No	18 (78.3)	14 (100)	
Yes	5 (21.7)	0 (0)	
**Nasal localization**			0.006
Turbinates	2 (8.7)	4 (28.6)	
Diffuse	17 (73.9)	3 (21.4)	
Septum	4 (17.4)	7 (50)	
**Number of affected nasal structure**			0.012
Multiple	16 (69.6)	3 (21.4)	
Single	7 (30.4)	11 (78.6)	
**Grouped appearance of the nasal lesion**			0.489
Crusted	4 (17.4)	1 (7.1)	
Granular	0 (0)	1 (7.1)	
Infiltrative	3 (13)	3 (21.4)	
Mixed	16 (69.6)	9 (64.3)	
**Intensity**			0.092
Severe	21 (91.3)	10 (71.4)	
Moderate	2 (8.7)	1 (7.1)	
Mild	0 (0)	3 (21.4)	
**Sporotrichosis outcome**			0.279
Cure	12 (52.2)	12 (85.7)	
Under treatment	2 (8.7)	0 (0)	
Death	2 (8.7)	0 (0)	
Loss of follow-up	7 (30.4)	2 (14.3)	
**Total**	**23 (62.16)**	**14 (37.84)**	

HIV: human immunodeficiency virus.

The presence/elimination of crusts had a similar percentage in both groups (57.1% versus 56.5%), the same occurring in relation to the "mixed" appearance of mucosal lesions (64.3% versus 69.6%). Septal perforation occurred exclusively in patients with the disseminated forms of the disease (n = 5/23; 21.7%) and the intensity of lesion presentation was higher in this group (91.3% versus 71.4%), despite the lack of statistical significance in both categories.

Regarding the outcome of sporotrichosis, considering the general context of the disease, the group with disseminated forms of sporotrichosis had a lower cure rate (52.2% versus 85.7%), but without statistical significance. The median (85 weeks; IQR: 60–146 weeks) of the treatment time for the cured patients in this group was greater than the median (28 weeks; IQR 19–45 weeks) of the treatment time required to achieve healing in the group with localized nasal mucosa (p = 0.01).

### Relative risks and survival analysis

**Fig 4:**
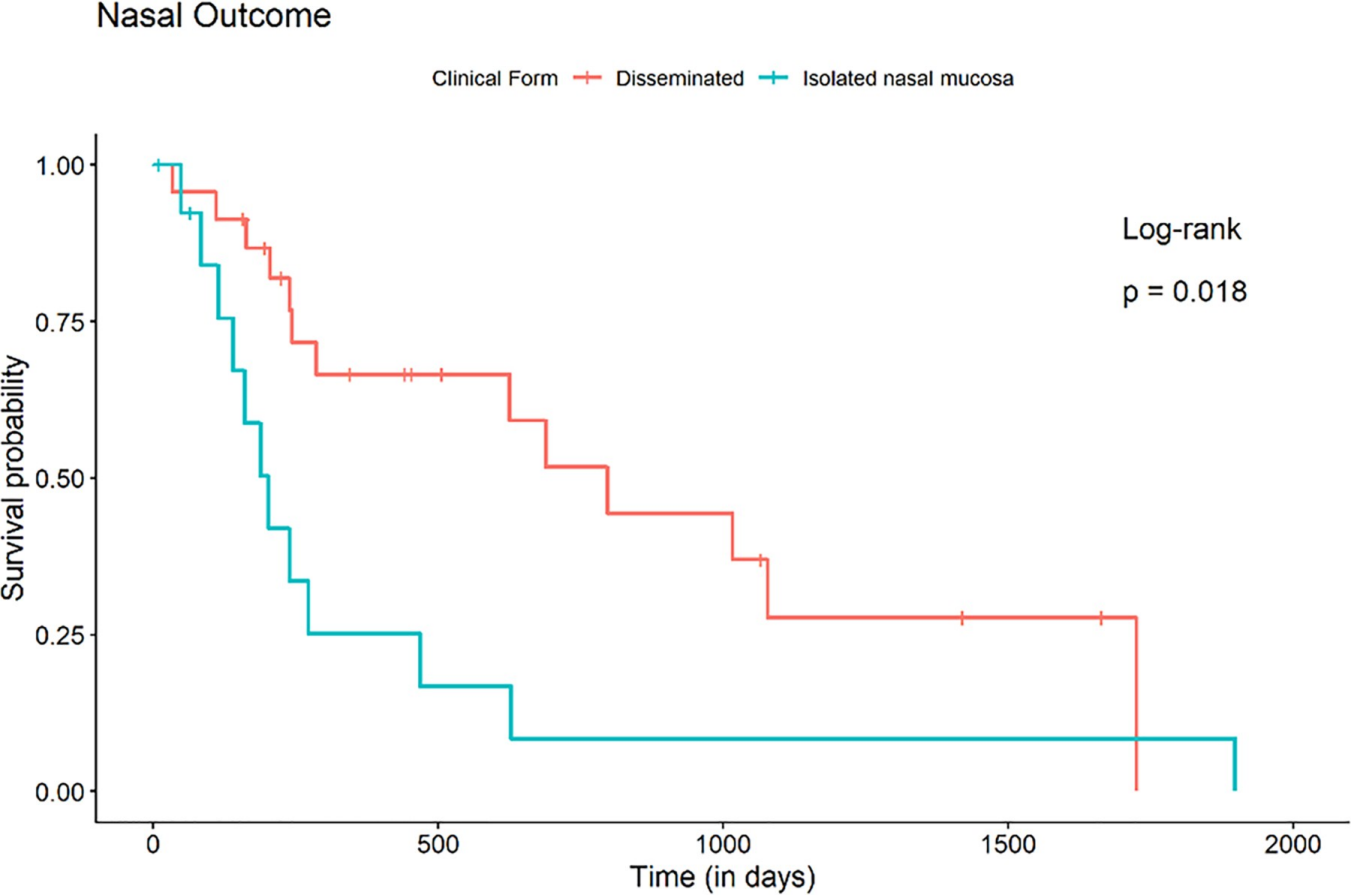
Comparative analysis of cure (Kaplan-Meier survival curve) between patients with isolated nasal mucosa sporotrichosis’ lesion and patients with the disseminated form.

Analyzing the groups by clinical form, the aHR of a patient with a localized nasal mucosa to achieve mucosal cure was 2.725 (95% CI: 0.859–8.639; p = 0.089) times that of a patient with a disseminated form. In addition, the aHR to achieve total cure of sporotrichosis was 6.16 (95% CI: 1.657–22.905; p <0.01) compared to a patient with disseminated sporotrichosis (in the disseminated forms there are other sites to cure) (**Fig 4**).

**Fig 5:**
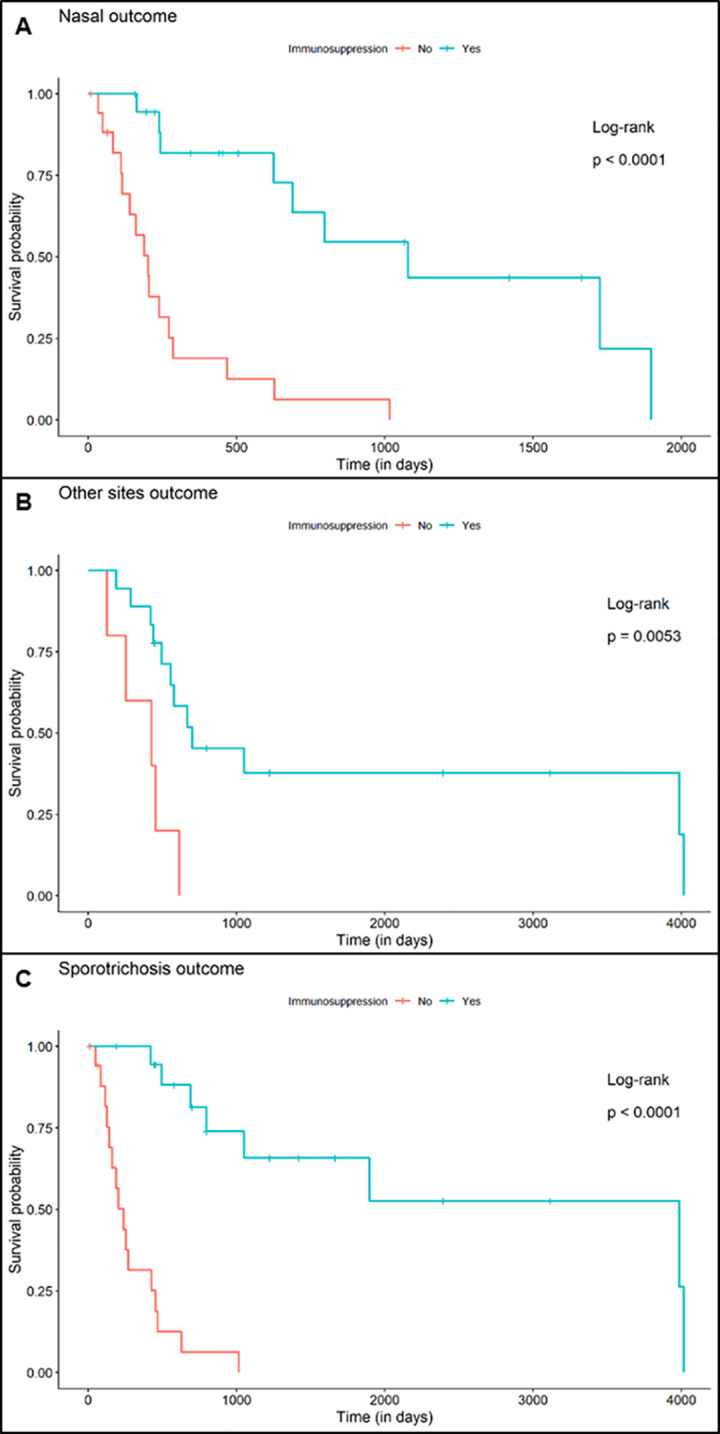
Comparative analysis of cure (Kaplan-Meier survival curves) in patients with and without immunosuppression. (A) Patients with sporotrichosis lesions in the nasal mucosa (Nasal outcome). (B) Patients with sporotrichosis lesions in other sites outcome, and in the general context (Sporotrichosis outcome).

When the patients were grouped as immunosuppressed or not, the aHR calculated for a patient without immunosuppression to achieve healing of nasal mucosa lesions was 8.628 (95% CI: 2.595–28.687; p <0.01) times that of a patient with immunosuppression. In addition, the aHR to achieve healing of the lesions in the other sites was 11.419 (95% CI: 1.992–65.575; p <0.01), and the total cure of sporotrichosis was 18.089 (95% CI: 4.045–80.894; p <0.01). This division was the most significant in showing increased risk between the groups (**Fig 5**).

Patients without HIV infection achieved cure of the lesions in the nasal mucosa with an aHR of 2.199 (95% CI: 0.602–8.036; p = 0.233) when compared to PLHIV. For the non-mucosal lesions, the aHR to cure was 5.127 (95% CI: 0.923–28.489; p = 0.062), and for the cure of sporotrichosis in a general context, the aHR was 6.595 (95% CI: 1.14–38.164; p = 0.035).

## Discussion

Before 1998, the average annual number of human sporotrichosis cases treated at INI/FIOCRUZ was 1.2 cases/year [1,2]. After this period, with the establishment of the hyperendemic of zoonotic transmission in the state of Rio de Janeiro and the consolidation of the INI/FIOCRUZ as the main reference center in the care and research of sporotrichosis cases, involvement of the nasal mucosa also became more frequent, with the present case series representing 0.7% of the institutional cases of sporotrichosis. For instance, in 2011, the peak of cases diagnosed at our institution, more than 600 individuals were diagnosed [28]. As sporotrichosis was not a notifiable disease in Rio de Janeiro state until 2013, it is difficult to know the real incidence and distribution of the disease in the city and state of Rio de Janeiro throughout the complete study period.

Comparing this case series to others of zoonotic transmission of sporotrichosis [29], there was a slight predilection for males and a slight reduction in the median age [1,2,17,28–30]. This may be due to cases in PLHIV, which in Brazil are mostly men, especially young adults [7,10,14,28], followed by adolescents and children (5 in total); the latter group often affected in the face [6]. The concentration of cases in the metropolitan region of Rio de Janeiro and in the north and west zones of the capital follow the pattern of the hyperendemic of zoonotic transmission [2]. Possible reasons for this concentration are its larger population and the location of the INI/FIOCRUZ in the city of Rio de Janeiro.

Regarding occupations, retirees and students were equally affected in this study. This second group was also one of the most common in a previous investigation [2]. Involvement in domestic activities, proximity and care of domestic cats with sporotrichosis may explain the higher frequency of both groups [2,29]. Interestingly, housewives were not highlighted in this sampling, which differs to the general sporotrichosis cases published in this region [1,2,29].

The classical environmental form of fungal transmission [1] was not predominant in this study. The likely main transmission forms reported by patients restricted to the nasal mucosa (local digital trauma, direct contact and/or proximity to infected cats) reinforce the zoonotic transmission that occurs in the state of Rio de Janeiro [1,2]. Interestingly, in one patient report, the probable transmission occurred after sharing a nasal spray with the cat, who was also sick. Cats have a high fungal burden in their lesions [2,31] and a high frequency of nasal involvement [16,31], which implies a worse prognosis due to feeding and breathing difficulties [16]. In addition, their sneezing and the exudation/hypersecretion of their lesions may increase the propagation of particles containing *Sporothrix* sp. through aerosols, contributing to the infection of people living with them [16]. The possibility of transmission of *S*. *brasiliensis* through the sneezes of cats with sporotrichosis was verified in a recent study [3].

The most diagnosed clinical presentations of sporotrichosis worldwide are lymphocutaneous and fixed cutaneous [6], both localized forms of the disease. Disseminated and disseminated cutaneous forms are less frequent and usually associated with immunosuppression or comorbidities [7–11,14]. This study showed that, in addition to the increase in the total number of sporotrichosis cases, there was also an increase in the diagnosis of nasal mucosa involvement (disseminated/extracutaneous form), which occurred alone in 14 (37.84%) cases.

There were cases of association of nasal mucosa involvement with disseminated skin lesions or even with other foci of sporotrichosis dissemination. The latter, considered more severe [7–11,14], was observed in most cases, especially in PLHIV, reinforcing the association between these two conditions, as demonstrated in previous studies [10,14]. The classification of the clinical forms of sporotrichosis has undergone some variations over the years. Perhaps the multiplicity of modes of infection and dissemination (self-inoculation, contiguity, hematogenous or inhalation) contributes to the difficulty in classifying the mucosal forms.

Hematogenous dissemination, which usually affects immunosuppressed patients [7,8,10,12,14], was the probable route for the disseminated cases in this study, largely composed of people with comorbidities, notably PLHIV. In contrast to this first group, only two cases restricted to the nasal mucosa exhibited comorbidities, which suggests, for this second group, inhalation or direct inoculation in the nasal mucosa [2].

In the nose, cutaneous lesions eventually appear on the skin of the dorsum or tip [8,9,32,33]. The extension to the nasal mucosa and other mucous membranes of the upper aerodigestive tract, as well as their clinical manifestations, are poorly described in the literature and are more commonly reserved for the disseminated and disseminated cutaneous forms of the disease [2,8,9,14,19–21]. Some signs and symptoms found in this study, such as nasal congestion, mucopurulent secretion, progressive nasal obstruction, nasal bleeding, PNS mucosal involvement, bone erosion and septal perforation, are also frequently described in the literature [8,19–21].

It is worth noting that even in cases with mild nasal signs and symptoms, such as rhinorrhea, hyperemia, synechia and dryness, which may not be reported by patients, we observed the presence of nasal mucosa lesions. In contrast, skin lesions, especially visible ones, which are eventually intense and unaesthetic, are of greater concern to patients and are therefore more frequently reported [5].

The clinical presentations of sporotrichosis lesions in the nasal mucosa seen in this study included infiltrations and crusts that mainly affected the anterior nasal septum, in addition to granular and granular with microabscesses aspects. In this study, the mixed aspect (infiltration, crusts, microabscesses etc.) and severe lesion intensity predominated. Although uncommon in sporotrichosis, there were cases of destruction/perforation of the nasal septum, a condition more frequent in other granulomatous diseases, such as leprosy, tuberculosis, leishmaniasis and syphilis [14,34]. Specifically, for case 28, the extent of nasal septum destruction caused a flattening of the nasal pyramid.

The isolation and identification of *Sporothrix* spp. from the samples obtained from sporotrichosis lesions are the gold standard in the laboratory diagnosis of the disease [2] and were inclusion criteria in this study. In most cases, sample was obtained by nasal lesion swab/scraping, a relatively simple test with low costs and risks, compared to biopsy, which is more invasive [6]. Biopsy can help in the differential diagnosis, including tumors and non-granulomatous inflammatory diseases, especially when fungal structures are seen, increasing the likelihood of the diagnosis of sporotrichosis, or by visualizing other agents or inflammatory processes, such as amastigotes in leishmaniasis, bacilli in leprosy or tuberculosis or granulomas characteristic of sarcoidosis. The confirmation of the isolates molecularly identified as *S*. *brasiliensis* corroborates the zoonotic transmission and its association with this species in the hyperendemic ongoing since the late 1990s in the state of Rio de Janeiro, with more severe and atypical cases [30].

Maxillary sinusitis associated with sporotrichosis is poorly described in the literature [19–21]. Changes in the sinus mucosa and PNS content, erosion of the sinus walls and perforation of the nasal septum were also identified in other reports [19–21]. Although there are similarities in these imaging findings, there are limitations to affirm that the changes found in the PNS in this case series were directly caused by sporotrichosis because samples for etiological investigation were not collected specifically for this site. A secondary bacterial process, due to difficulty in draining the PNS, after inflammation of the nasal mucosa resulting from sporotrichosis, is a hypothesis to be considered.

Itraconazole is the first option for the sporotrichosis treatment due to its efficacy, safety and dosage convenience [6], even when administered in low doses [22]. In this case series, it was widely administered alone or, in moderate and severe cases, in combination with other antifungals, simultaneously or sequentially. Notably, in some cases, even after complete remission of the skin lesions, nasal lesions persisted, suggesting the maintenance of a perivascular infiltration process during the granulomatous phase of the disease, similar to what is observed in some granulomatous diseases, such as leprosy and leishmaniasis [34,35].

The clinical cure of patients with localized cutaneous sporotrichosis is achieved in approximately two to three months [6]. Compared to the nasal mucosa, some hypotheses to explain the increase in the median treatment time observed in this study are: a) the indiscriminate use of topical vasoconstrictor drugs that cause superficial ulceration and epistaxis and may lead to septal perforation; b) capillary fragility resulting from the use of regular inhaled medications, hypertension and/or hormonal changes; c) intense vasculitis that mainly affects the anterior septal region (Kiesselbach area) and predisposes the patient to nasal bleeding; d) mechanical trauma in the attempt to eliminate nasal crusts [34] and e) the large percentage of comorbidities, such as HIV/AIDS, compromising patients’ immune systems. An exacerbated cellular response can also cause injury or accentuate damage to the nasal mucosa [18].

Although a longer treatment time and drug combinations were needed, a good cure rate was achieved, even in a group of patients with immunosuppression, clinical severity, and dissemination. A dose of 100 mg/day of itraconazole was able to cure some of the cases of isolated nasal mucosa involvement. Even though this cure rate was satisfactory, the comparison of this outcome between patients with the localized nasal mucosa and those with the disseminated forms reflects the difficulties faced in the resolution of the cases that comprise the latter group, represented mainly by the immunosuppressed, especially PLHIV. These are also responsible for the highest number of hospitalizations and deaths, as previously published [10,15].

The great loss of follow-up observed in this sample may be associated with the severity of many cases, with prolonged hospitalizations and progression to death, sometimes with little importance given to periodic ENT evaluation, due to cases involving meningitis, for example. This loss, associated with the large variation found in the treatment time of patients, confers limitations to the study, which is characteristic of a retrospective design. Although this is a relatively large case series, it is not enough for some statistical analysis.

The systematization of the ENT examination with nasal endoscopy in patients with sporotrichosis may optimize tracking of mucosal lesions. Patients with sporotrichosis and facial lesions, lesions in more than one anatomical site (disseminated forms), immunosuppression or nasal complaints, even those mild ones, should be evaluated by an ENT specialist. The early identification of lesions can directly influence the time of treatment and improve cure rate. In addition, instituting local hygiene measures to prevent secondary infection and/or bleeding may minimize esthetic and functional sequelae.

This relatively large case series on a rare type of presentation of sporotrichosis demonstrates the importance of the subject and may contribute to the management of nasal sporotrichosis in the future.

## Supporting information

S1 STROBE Statement–Checklist of items that should be included in reports of observational studies. (DOCX)

S1 Table Definition of variables analyzed in Cox models. (DOCX)

S2 Table Sociodemographic and epidemiological data of 37 cases of sporotrichosis in the nasal mucosa treated at the ENT outpatient clinic of the INI/FIOCRUZ (1998–2020). (DOCX)

S1 Fig Annual series of sporotrichosis in the nasal mucosa treated at the ENT outpatient clinic of the INI/FIOCRUZ (1998–2020). Source: Electronic patient data system and Laboratory of Mycology database, both from INI/FIOCRUZ.
(TIF)

S2 Fig Agarose (1%) gel electrophoresis demonstrating the molecular identification of three clinical isolates of *Sporothrix brasiliensis*. Representative Agarose (1%) gel electrophoresis for *Sporothrix* species identification. Amplification of a DNA sequence of three clinical isolates using species-specific primers. (1, 7, 13 and 19) Molecular weight 1 kb, (2) Positive DNA control for *S*. *brasiliensis* (IPEC 16490), (3, 9 and 15) IPEC 52482, (4, 10 and 16) IPEC 51394, (5, 11 and 17) IPEC 44761, (6, 12 and 18) Negative control (no *Sporothrix* sp. DNA), (8) Positive DNA control for *S*. *schenckii* (IPEC 36275), (14) Positive DNA control for *S*. *globosa* (IPEC 27135). Source: Anna Carolina Procópio-Azevedo (Laboratory of Mycology, INI/FIOCRUZ).
(TIF)
