# Supplementary material for: Sporotrichosis in the nasal mucosa: A single-center retrospective study of 37 cases from 1998 to 2020
Source: PLoS Negl Trop Dis. 2023 Mar 27;17(3):e0011212. doi: 10.1371/journal.pntd.0011212 (PMC10079221; doi:10.1371/journal.pntd.0011212)
Supplement: S1 STROBE — (DOCX) [file pntd.0011212.s001.docx]

**S1 STROBE Statement – Checklist of items that should be included in reports of observational studies**

|  | **Item No** | **Recommendation** |
| --- | --- | --- |
| **Title and abstract** | 1 | (*a*) Indicate the study’s design with a commonly used term in the title or the abstract. The term is indicated in both the title (retrospective study) and in the abstract (lines 65-6: “Data were reviewed from the medical records and stored in a database”). |
|  |  | (*b*) Provide in the abstract an informative and balanced summary of what was done and what was found. This is done in the abstract. |
| **Introduction** | | |
| Background/rationale | 2 | Explain the scientific background and rationale for the investigation being reported. Available with relevant references. |
| Objectives | 3 | State specific objectives, including any prespecified hypotheses. Available in lines 62-5: “This study aimed to describe the epidemiological, clinical, and therapeutic profiles of 37 cases of sporotrichosis with involvement of the nasal mucosa…”. |
| **Methods** | | |
| Study design | 4 | Present key elements of study design early in the paper. Available in lines 174-5: “The records of the Laboratory of Mycology and of the ENT outpatient clinic, both from INI/FIOCRUZ, were used”. |
| Setting | 5 | Describe the setting, locations, and relevant dates, including periods of recruitment, exposure, follow-up, and data collection. Available in methods topics (p. 07-11). |
| Participants | 6 | (*a*) *Cohort study*—Give the eligibility criteria, and the sources and methods of selection of participants. Describe methods of follow-up. Available in methods topics (p. 07-11).  *Case-control study*—Give the eligibility criteria, and the sources and methods of case ascertainment and control selection. Give the rationale for the choice of cases and controls  *Cross-sectional study*—Give the eligibility criteria, and the sources and methods of selection of participants |
|  |  | (*b*) *Cohort study*—For matched studies, give matching criteria and number of exposed and unexposed. Not applicable.  *Case-control study*—For matched studies, give matching criteria and the number of controls per case |
| Variables | 7 | Clearly define all outcomes, exposures, predictors, potential confounders, and effect modifiers. Give diagnostic criteria, if applicable. Available in methods topics (p. 07-11). |
| Data sources/ measurement | 8* | For each variable of interest, give sources of data and details of methods of assessment (measurement). Describe comparability of assessment methods if there is more than one group. Available in methods topic “Analysis of collected data” (lines 238-64). |
| Bias | 9 | Describe any efforts to address potential sources of bias. Partially addressed in the “Analysis of collected data” (lines 238-64) and later in the “Discussion” section |
| Study size | 10 | Explain how the study size was arrived at. This was an institutional cohort, with its size obtained after the review of cases who fulfilled an inclusion criteria, a convenience sample. This explanation is given in lines 172-7. |
| Quantitative variables | 11 | Explain how quantitative variables were handled in the analyses. If applicable, describe which groupings were chosen and why. Available in methods topic “Analysis of collected data” (lines 238-64). |
| Statistical methods | 12 | (*a*) Describe all statistical methods, including those used to control for confounding. Available in methods topic “Analysis of collected data” (lines 238-64). |
|  |  | (*b*) Describe any methods used to examine subgroups and interactions. Available in methods topic “Analysis of collected data” (lines 238-64). |
|  |  | (*c*) Explain how missing data were addressed. “The terms “not available”, “no epidemiological history”, and “unknown” are considered missing data” (lines 261-2). |
|  |  | (*d*) *Cohort study*—If applicable, explain how loss to follow-up was addressed. Loss to follow-up are considered in outcome variable (lines 254-5)  *Case-control study*—If applicable, explain how matching of cases and controls was addressed  *Cross-sectional study*—If applicable, describe analytical methods taking account of sampling strategy |
|  |  | (*e*) Describe any sensitivity analyses. Not applicable. |
| **Results** |  |  |
| Participants | 13* | (a) Report numbers of individuals at each stage of study—eg numbers potentially eligible, examined for eligibility, confirmed eligible, included in the study, completing follow-up, and analysed. Available in results topic “Demographic aspects” (line 268-73). |
| Participants | 13* | (a) Report numbers of individuals at each stage of study—eg numbers potentially eligible, examined for eligibility, confirmed eligible, included in the study, completing follow-up, and analysed. Available in results topic “Demographic aspects” (line 268-73). |
|  |  | (b) Give reasons for non-participation at each stage. This study was designed in a single stage and all included participants took part in it. |
|  |  | (c) Consider use of a flow diagram. We opted not to use a diagram, due to the simple numbers. |
| Descriptive data | 14* | (a) Give characteristics of study participants (eg demographic, clinical, social) and information on exposures and potential confounders. Available in results topics (lines 269-428). |
|  |  | (b) Indicate number of participants with missing data for each variable of interest. . “The terms “not available”, “no epidemiological history”, and “unknown” are considered missing data” (lines 261-2), and are shown in the tables. |
|  |  | (c) *Cohort study*—Summarise follow-up time (eg, average and total amount). Available in results topic “Treatment: medication used, time and outcome” (lines 359-79). |
| Outcome data | 15* | *Cohort study*—Report numbers of outcome events or summary measures over time. Available in results topic “Treatment: medication used, time and outcome” (lines 359-79). |
|  |  | *Case-control study—*Report numbers in each exposure category, or summary measures of exposure |
|  |  | *Cross-sectional study—*Report numbers of outcome events or summary measures |
| Main results | 16 | (*a*) Give unadjusted estimates and, if applicable, confounder-adjusted estimates and their precision (eg, 95% confidence interval). Make clear which confounders were adjusted for and why they were included. Available in results topics “Comparison between patients with localized nasal mucosa and patients with the form associated with other sites (disseminated)” and “Relative risks and survival analysis” (lines 385-428). |
|  |  | (*b*) Report category boundaries when continuous variables were categorized. For this study, the continuous variables were not categorized. |
|  |  | (*c*) If relevant, consider translating estimates of relative risk into absolute risk for a meaningful time period. Not relevant. |
| Other analyses | 17 | Report other analyses done—eg analyses of subgroups and interactions, and sensitivity analyses. Available in results topics “Comparison between patients with localized nasal mucosa and patients with the form associated with other sites (disseminated)” (lines385-408). |
| **Discussion** | | |
| Key results | 18 | Summarise key results with reference to study objectives. Available all over the discussion topic. |
| Limitations | 19 | Discuss limitations of the study, taking into account sources of potential bias or imprecision. Discuss both direction and magnitude of any potential bias. Available in discussion topic (lines 567-70). |
| Interpretation | 20 | Give a cautious overall interpretation of results considering objectives, limitations, multiplicity of analyses, results from similar studies, and other relevant evidence. Available all over the discussion topic. |
| Generalisability | 21 | Discuss the generalisability (external validity) of the study results. Available in lines 575-85. |
| **Other information** | | |
| Funding | 22 | Give the source of funding and the role of the funders for the present study and, if applicable, for the original study on which the present article is based. Available in funding topic (lines 593-8). |
