# Supplementary material for: Sporotrichosis in the nasal mucosa: A single-center retrospective study of 37 cases from 1998 to 2020
Source: PLoS Negl Trop Dis. 2023 Mar 27;17(3):e0011212. doi: 10.1371/journal.pntd.0011212 (PMC10079221; doi:10.1371/journal.pntd.0011212)
Supplement: S1 Table — (DOCX) [file pntd.0011212.s002.docx]

**S1 Table. Definition of variables analyzed in Cox models.**

| **Variable** | **Variable definition** |
| --- | --- |
| **Outcome^1^** | |
| Nasal mucosa (localized) | Lesion restricted to the nasal mucosa or accompanied by contiguous and/or reactional lesions |
| Other sites | All other sporotrichosis lesions, not in the nasal mucosa |
| Sporotrichosis | All sporotrichosis lesions - nasal mucosa or not |
| **Exposure** | |
| Nasal mucosa (localized) clinical form x Disseminated clinical form | Lesion restricted to the nasal mucosa  x Nasal mucosa lesion associated with other sites |
| Immunosuppression | Yes or No |
| HIV infection | Yes or No |
| **Adjustment (control)** | |
| Age | Median and IQR |
| Sex | Male or Female |
| Septal perforation | Yes or No |
| Number of affected nasal structures | Single x Multiple |

HIV: human immunodeficiency virus; IQR: interquartile range. ^1^Outcomes assessed were cure, under treatment, or loss of follow-up.
