# Supplementary material for: Sporotrichosis in the nasal mucosa: A single-center retrospective study of 37 cases from 1998 to 2020
Source: PLoS Negl Trop Dis. 2023 Mar 27;17(3):e0011212. doi: 10.1371/journal.pntd.0011212 (PMC10079221; doi:10.1371/journal.pntd.0011212)
Supplement: S2 Table — (DOCX) [file pntd.0011212.s003.docx]

**S2 Table. Sociodemographic and epidemiological data of 37 cases of sporotrichosis in the nasal mucosa treated at the ENT outpatient clinic of the INI/FIOCRUZ (1998-2020).**

| Case | Sex /Age  (years) | Municipality | Profession / occupation | Probable transmission |
| --- | --- | --- | --- | --- |
| 1 | M / 34 | Rio de Janeiro | Unemployed | No epidemiological history |
| 2 | M / 31 | São Gonçalo | Unemployed | Infected cat |
| 3 | F / 66 | Belford Roxo | Merchant | Infected cat |
| 4 | M / 38 | Rio de Janeiro | Teacher | Soil, plants and/or organic matter |
| 5 | M / 37 | Rio de Janeiro | Technical professional | Infected cat |
| 6 | F / 44 | Rio de Janeiro | Teacher | Infected cat |
| 7 | F / 64 | São João de Meriti | Retiree | No epidemiological history |
| 8 | M / 38 | Rio de Janeiro | Fitter | Infected cat |
| 9 | F / 56 | Rio de Janeiro | NA | Infected cat |
| 10 | M / 33 | Rio de Janeiro | Accounting assistant | Infected cat |
| 11 | F / 41 | São Pedro da Aldeia^1^ | Retiree | Soil, plants and/or organic matter |
| 12 | F / 68 | Duque de Caxias | Retiree | Infected cat |
| 13 | M / 38 | Rio de Janeiro | Bricklayer | Infected cat |
| 14 | M / 44 | Nova Iguaçu | Unemployed | Infected cat |
| 15 | F / 36 | Rio de Janeiro | Housekeeper | Infected cat |
| 16 | M / 69 | Duque de Caxias | Retiree | Infected cat |
| 17 | F / 49 | Rio de Janeiro | NA | Infected cat |
| 18 | M / 52 | Duque de Caxias | Painter | Infected cat |
| 19 | F / 47 | Rio de Janeiro | Attendant | Infected cat |
| 20 | M / 37 | Rio de Janeiro | Hairdresser | Infected cat |
| 21 | M / 35 | Rio de Janeiro | General services assistant | No epidemiological history |
| 22 | M / 63 | Rio de Janeiro | Retiree | Infected cat |
| 23 | F / 25 | Nova Iguaçu | Technical professional | Infected cat |
| 24 | F / 48 | Rio de Janeiro | Cook | Infected cat |
| 25 | F / 11 | Rio de Janeiro | Student | Infected cat |
| 26 | M / 11 | Duque de Caxias | Student | No epidemiological history |
| 27 | M / 43 | Duque de Caxias | Painter | No epidemiological history |
| 28 | M / 44 | Duque de Caxias | General services assistant | Infected cat |
| 29 | F / 17 | Duque de Caxias | Student | Infected cat |
| 30 | F / 41 | Nilópolis | Merchant | Infected cat |
| 31 | F / 36 | Rio de Janeiro | Administrative Assistant | Infected cat |
| 32 | F / 44 | Rio de Janeiro | Housekeeper | No epidemiological history |
| 33 | M / 46 | Itaboraí | Bricklayer | Soil, plants and/or organic matter |
| 34 | M / 21 | Rio de Janeiro | Administrative Assistant | Infected cat |
| 35 | F / 10 | Rio de Janeiro | Student | Infected cat |
| 36 | M / 30 | Rio de Janeiro | Veterinary Doctor | Infected cat |
| 37 | M / 16 | Rio de Janeiro | Student | Infected cat |

M: Male; F: Female; ^1^ São Pedro da Aldeia was the only city outside the metropolitan region of Rio de Janeiro. NA: Not available. Source: Electronic patient data system and Laboratory of Mycology database, both from INI/FIOCRUZ.
