# Supplementary figures and images for: Sporotrichosis in the nasal mucosa: A single-center retrospective study of 37 cases from 1998 to 2020
Source: PLoS Negl Trop Dis. 2023 Mar 27;17(3):e0011212. doi: 10.1371/journal.pntd.0011212 (PMC10079221; doi:10.1371/journal.pntd.0011212)

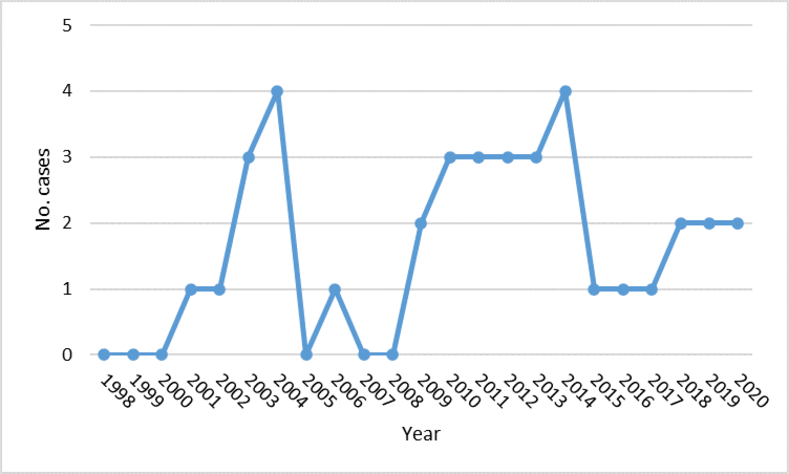

Supplement: S1 Fig — Source: Electronic patient data system and Laboratory of Mycology database, both from INI/FIOCRUZ. (TIF) [file pntd.0011212.s004.tif]

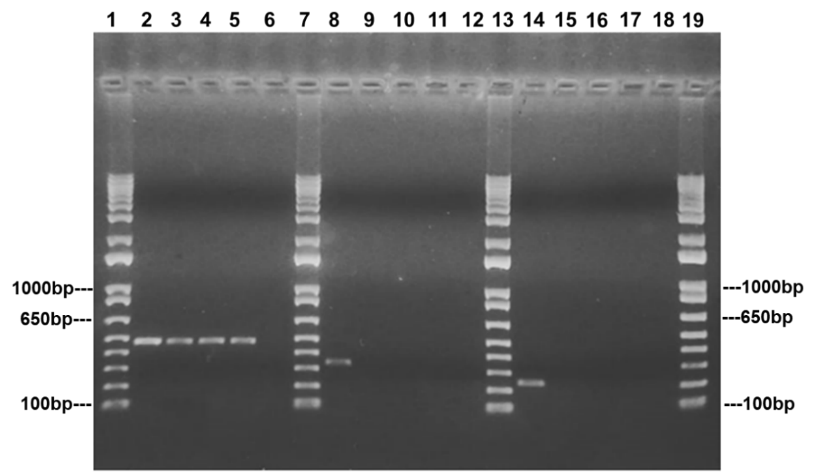

Supplement: S2 Fig — Representative Agarose (1%) gel electrophoresis for Sporothrix species identification. Amplification of a DNA sequence of three clinical isolates using species-specific primers. (1, 7, 13 and 19) Molecular weight 1 kb, (2) Positive DNA control for S. brasiliensis (IPEC 16490), (3, 9 and 15) IPEC 52482, (4, 10 and 16) IPEC 51394, (5, 11 and 17) IPEC 44761, (6, 12 and 18) Negative control (no Sporothrix sp. DNA), (8) Positive DNA control for S. schenckii (IPEC 36275), (14) Positive DNA control for S. globosa (IPEC 27135). Source: Anna Carolina Procópio-Azevedo (Laboratory of Mycology, INI/FIOCRUZ). (TIF) [file pntd.0011212.s005.tif]
